# Feasibility of a Low-Intensity Task-Shared Intervention for Common Mental Disorders in Survivors of Intimate Partner Violence in India: A Mixed Methods Study

**DOI:** 10.1177/10778012251351898

**Published:** 2025-06-23

**Authors:** Abhijit Nadkarni, Devika Gupta, Kedar Mirchandani, Anushka Patel, Shahzaad Hussain, Marimilha Grace Pacheco, Shruti Bora, Arista Jhanjee, Shreya Sharma, Jasmine Kalha, Kaustubh Joag, Urvita Bhatia

**Affiliations:** 1 Centre for Global Mental Health, Department of Population Health, 4906 London School of Hygiene and Tropical Medicine, London, UK; 2 Addictions and Related Research Group (ARG), 561105 Sangath, Goa, India; 3 Department of Psychiatry, Massachusetts General Hospital, Boston, MA, USA; 4 Centre for Mental Health Law and Policy, 534334 Indian Law Society, Pune, India

**Keywords:** acceptability, feasibility, task-sharing, common mental disorders, intimate partner violence, India

## Abstract

Our mixed-methods study evaluated the acceptability and feasibility of a lay-counselor-delivered low-intensity psychosocial intervention for women who experienced intimate partner violence in India. We found statistically significant improvements in mental health outcomes like depression and anxiety. Participants also reported positive changes in thoughts, behavior, emotional management, relationships, self-care, and daily functioning. The use of lay-counselors offers a scalable solution in resource-limited settings, addressing a critical need in low- and middle-income countries. This approach highlights a novel and effective strategy for supporting IPV survivors in culturally diverse contexts.

## Introduction

While the World Health Organization (WHO) defines domestic violence (DV) as acts of physical, sexual, or psychological abuse, as well as control by an intimate partner, it acknowledges that DV could include behaviors perpetrated by other members of a household in some countries (Kalokhe et al., 2015). Intimate partner violence (IPV) is a form of DV that occurs between current or former spouses. Globally, 26–28% of ever-married/partnered women between 20 and 44 years have been subjected to IPV at least once in their lifetime (WHO, 2021). The lifetime prevalence of physical and/or sexual IPV among women aged 15–49 years is highest among the “Least Developed Countries” (37%), followed by the regions of Southern Asia (35%) and Sub-Saharan Africa (33%) (WHO, 2021). India is one of 16 countries which fall within the second highest prevalence range (35–39%) of women aged 15–49 experiencing physical and/or sexual IPV at least once in their lifetime (WHO, 2021).

The mental health impact of IPV includes post-traumatic stress disorder (PTSD), depression, suicidal ideation and attempts, anxiety, and substance abuse disorders (Insan et al., 2022; Koirala & Chuemchit, 2020; Ogden et al., 2022; Spencer et al., 2022; White et al., 2023). Outside of clinical diagnostic categories, IPV results in several other adverse psychosocial effects, such as fear, shame, stigma, social isolation, economic hardship, and concern for one's children (Wessells & Kostelny, 2022). Some of these are particularly exacerbated in low- and middle-income countries (LMICs), which might have greater economic stresses, limited health and social services, armed conflicts, breakdown of law and order, and cultural practices such as dowry deaths and honour killings of women (Wessells & Kostelny, 2022). Compared to the general population, Indian women who experience IPV (a) have a higher likelihood of experiencing depression, PTSD, and attempted suicide, (b) report higher frequencies of injuries, chronic diseases such as asthma and anemia, gynecologic morbidity, and infectious diseases such as sexually transmitted infections and HIV, and (c) are more likely to experience terminated unintended pregnancies and utilize less prenatal care and breastfeeding (Kalokhe et al., 2015). Additionally, their children have a greater probability of being malnourished, developing asthma, being insufficiently vaccinated, and dying early (Kalokhe et al., 2015).

In India, only one of every seven women who experience IPV seeks help from anyone, and less than 1% seek help from formal institutions for several reasons, including patriarchal social norms and stigma related to disclosing IPV (Dehingia et al., 2022). Additionally, health providers in India do not receive training specific to IPV responses, and survivors lack information about and access to appropriate formal resources for IPV. Formal institutions might not acknowledge, validate and respectfully respond to the survivors’ needs, especially related to their mental health (Bhattacharyya, 2015; Mahapatro & Gupta, 2014; Majumdar, 2004). These barriers reflect those in other LMICs, where survivors of IPV do not access help because of fear of divorce, stigmatization, lack of trust in formal support services, sociocultural norms emphasizing gender role expectations, and family privacy (Rohn & Tenkorang, 2024).

Several psychological interventions have been developed to directly address mental health problems among survivors of IPV, in addition to a range of secondary and tertiary psychosocial IPV interventions that also report positive effects on the mental health of survivors (Yapp et al., 2020). Some key underlying features of effective interventions include elements of problem-solving, choice facilitation/goal-setting, techniques to alter negative thoughts of self, others, and the world, intensive advocacy (helping survivors to make sense of the situation, identify potential solutions, and achieve the goals they have set), and cognitive and cognitive-behavioral components (Trabold et al., 2020).

A substantial proportion of individuals experiencing IPV access routine health services for their general healthcare needs, and health practitioners are the major professional group to whom patients are likely to disclose IPV (WHO, 2013a, 2013b). Despite this, only a minority of individuals are screened and recognized as enduring IPV in health care settings; and when they do disclose, health professionals often lack necessary skills to respond appropriately (Feder et al., 2006; García-Moreno et al., 2015). In the absence of accessible specialty services to address IPV, many LMICs have implemented in-service training for general healthcare workers to address provider knowledge, attitudes and practice. However, there are persistent challenges related to high staff turnover, scaling up of training, inadequate responses for emotional or economic violence, and lack of privacy for survivors within health facilities (Sikder et al., 2021).

To summarize, in India the prevalence of IPV remains high, yet survivors often hesitate to seek help due to a variety of reasons outlined above. This lack of help-seeking underscores an urgent need for contextually relevant and accessible interventions. By leveraging community-based lay-counselor–delivered programs, it is possible to bridge gaps in formal services, reduce the psychological burden associated with IPV, and empower survivors to seek and receive the support they need.

To address these gaps, the aim of our program was to develop a contextually relevant intervention to respond to the mental health impact of IPV on women in India. Through a systematic intervention development process (described in a separate paper), we developed *Pahal* (“first step” in Hindi), a psychosocial intervention for IPV to be delivered in communities through task-sharing with nonspecialist workers. This study was guided by the UK MRC framework for developing and evaluating complex interventions (Skivington et al., 2021) and our extensive experience of developing culturally relevant psychosocial interventions in low resource settings (Nadkarni et al., 2014).

## Methods

The aim of this study was to evaluate the acceptability and feasibility of *Pahal*, delivered by lay-counselors to women who had experienced IPV.

### Design

Mixed-methods study. We conducted an uncontrolled treatment cohort with pre-post evaluation and a nested qualitative study.

### Settings

The state of Goa (semi-urban setting) and Mehsana district in the state of Gujarat (rural setting), both in Western India. Compared to other states in India, Goa ranks high on socio-economic parameters, with 62% of its population living in urban areas, a sex ratio of 973 females per 1000 males, and average literacy rate of 90%. Mehsana has a population of approximately two million adults, of which nearly half-live in rural areas. The sex ratio in this district is 926 females per 1000 males. The most recent estimates related to DV from the National Family Health Survey-5 reported that on average, 15% and 20% of ever-married women have experienced DV in Gujarat and Goa, respectively. In Mehsana, our program was nested within an existing community program called *Atmiyata*, a rural community-led intervention using community volunteers to identify and provide a package of community-based interventions for common mental disorders (CMDs) (Shields-Zeeman et al., 2017). *Atmiyata* community volunteers had reported a high prevalence of DV.

### Sample

We identified potential participants through convenience sampling using strategies described under “procedures” below. We included adult women (≥18 years) who experienced not more than six instances of physical or sexual violence perpetrated by their husband in the past six months, and who scored ≥6 on the 12-item General Health Questionnaire (GHQ-12) (Golderberg & Williams, 1988). The GHQ-12 is a screening tool to identify psychological distress (CMDs including depression and anxiety) and has been validated for use in Goa (Patel et al., 2008) and used in Mehsana (Joag et al., 2020).

Given that *Pahal* was a new low-intensity intervention delivered by nonspecialists, we did not want to use it as a response to severe or high frequency violence. Consistent with another study conducted in India that screened out for high-risk violence (Hartmann et al., 2021), we excluded women who had experienced (a) severe violence characterized by an attempt to choke, burn, threaten to use, or actually use a weapon in the previous six months; or (b) more than six instances of physical or sexual violence in the past six months. Additionally, we also excluded any potential participant who disclosed that participating in the study would increase their likelihood of experiencing violence. For Mehsana, we dropped the exclusion criterion related to frequency of violence because we observed that married women experiencing high frequency of IPV were being identified more commonly, possibly because of the existing strong community linkages of the Atmiyata program. Hence, there was a strong likelihood of those in most need being excluded from our study in a setting where usual care for survivors of IPV is nonexistent (which is not the case in Goa).

In all 38 women (26 in Goa, and 12 in Mehsana) consented to enter the study and completed the baseline assessment.

### Intervention

*Pahal* is a manualized intervention developed through an intensive intervention development process that included (a) in-depth interviews (IDIs) with survivors of IPV and service providers in Goa (where the intervention was first developed) to understand needs and experiences of service users; (b) a review of systematic reviews of effective interventions for IPV and international guidelines for IPV responders to identify intervention components; (c) consultative workshops with local stakeholders to delineate a Theory of Change for the intervention and identify priorities for intervention outcomes and delivery; and (d) a Delphi survey and intervention development group discussions with national and international experts in mental health and/or violence research to distil the final components and build consensus on the intervention.

Following this multistep process, *Pahal* was designed as a structured, manualized and modular intervention meant to be delivered in 6–8 sessions. The intervention was nested within an internal stepped-care model which allowed for participants with more severe symptoms or life problems to access higher-intensity services (e.g., assisted case management) and/or referral to a specialist provider for longer-term care.

**Figure 1.**
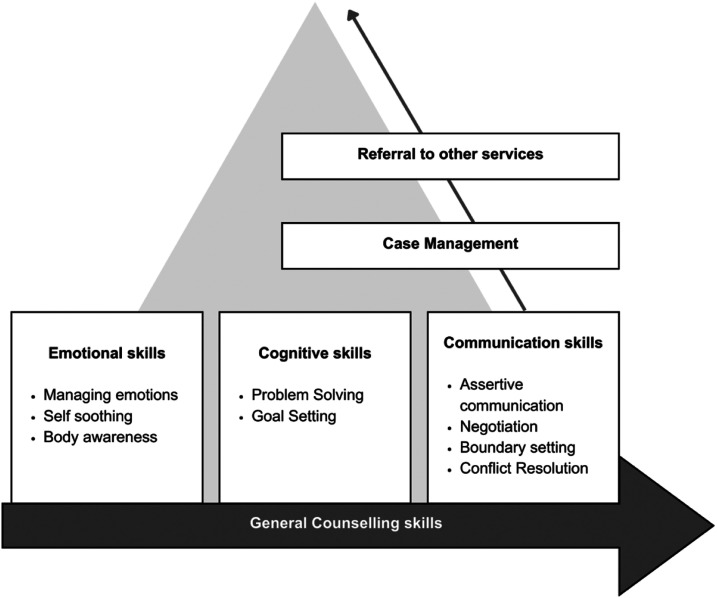
Conceptual Framework of *Pahal*.

The components of the *Pahal* intervention are summarized in Figure 1. All participants received measurement-based care (Aboraya et al., 2018) based on assessments conducted at the start of the counseling sessions, and at midline (between session numbers 3 and 5). Counselors were trained in selecting and matching intervention components based on the participant's needs (i.e., all participants did not always receive all components).

Counselors followed a continuous practice of weekly risk assessment for suicide or self-harm and escalation of violence, and conducted a thorough assessment if any risk was disclosed or suspected. Participants deemed to be at high risk of current or future violence, or who indicated the need for additional services, were connected to the team's case manager, who had strong links with the community and acted as a liaison with community resources and relevant referral agencies to facilitate referral to other service providers. We followed the same system in Mehsana, where the *Atmiyata* team members acted as case managers and renewed and leveraged their existing support and referral networks. Additionally, in Goa, detailed information about available services was shared with all counselors, who were trained to identify participants’ social needs and connect them to the appropriate referral agency via the case manager.

Participants were discharged after mutual agreement between counselor and client, or after treatment completion (6–8 sessions). Participants who did not respond to four consecutive efforts to be contacted and missed two consecutive scheduled sessions were considered as treatment dropouts.

### Delivery Agents

Potential lay counselors were adults (≥18 years), had completed at least high school education, had no professional qualification in the field of mental health, and were fluent in the vernacular languages in the study settings. In Goa, new individuals were recruited to be trained as counselors, and in Mehsana, existing *Atmiyata* “Champions” (referred to as counselors from here on) were recruited. They were local community volunteers trained to identify and provide a package of community-based interventions for persons with CMD (Pathare et al., 2020).

In Goa, we delivered a 40-hour training over 5 days covering general counseling skills and treatment-specific skills from *Pahal*. Training was delivered in English and the vernacular languages and led by psychologists. The training was multi-modal and included didactic lectures, roleplay-based skills demonstrations by the facilitators and roleplay-based skills practice for participants. Following training, the trainee competencies were assessed through structured roleplays rated for observable behaviors and skills on the ENhancing Assessment of Common Therapeutic (ENACT) factors scale (Kohrt et al., 2015). In Mehsana, since direct contact with the counselors was not feasible due to language constraints, we conducted the same training in a Train-the-Trainer format with *Atmiyata* Community Facilitators and team leads (collectively “supervisors”), who shared regular contact and long-term trust with the counselors. The supervisors then trained the counselors along with one member of our team involved to coach and observe.

In Goa, the newly trained counselors participated in supervision (4–6 hours per week) which included (a) weekly group supervision with case managers and specialists (i.e., clinical psychologists), (b) weekly peer-based supervision where they listened to audio-recorded sessions and provided each other with feedback, and (c) individual supervision once a month with expert psychologists. The counselors were provided with ratings and feedback by their supervisors and peers based on an audio recording of an individual counselor's recent session. A self-rating and supervisor rating on the ENACT counseling skills (“How skills”) and treatment-specific skills (“What skills”) were provided for each session based on the audio recording, and discussed in a group. In Mehsana, there was no acceptability of audio recording of sessions due to privacy concerns and a mistrust of technology, and low acceptability for pen-and-paper documentation. As a result, the counselors provided weekly updates to their supervisors, and attended group supervision meetings with them once in a fortnight. The supervisors updated an excel sheet based on these discussions and met the Goa team once a week to discuss questions and challenges, as well as record feedback to be conveyed to the counselors. At both sites, the supervision meetings provided a space for collaborative problem solving and planning: areas of skill deficit were identified and discussed, competencies were developed through role-plays, and next steps for each case were determined.

### Procedures

In Goa, participants were identified through referrals from community collaborators and gatekeepers such as Anganwadi workers (community health workers) and self-help group members. Participants were also identified via self-referrals that were triggered by awareness building activities in communities, including presentations at meetings of women's groups, WhatsApp messages and posters at community centers. In Mehsana, because counselors were from and had intimate knowledge of the communities in which they worked, they were able to directly identify potential participants from those communities. The program had also developed a community gatekeeper network that worked closely with different counselors to facilitate referrals into the program.

After verbal consenting, potential participants were screened for eligibility in person or on the phone. Eligible participants who provided informed written (witnessed thumbprint for illiterate participants) consent were enrolled in the study. Ineligible participants in Goa were referred on to psychosocial service providers based upon their need, while those in Mehsana were referred into *Atmiyata* for counseling.

### Measures

The following data were collected by trained field researchers at the baseline, midline (between sessions 3 and 5), and at the endline (eight weeks after the first counseling session):

- Nine item Patient Health Questionnaire (PHQ-9) (Kroenke et al., 2001) which assesses the frequency of depressive symptoms over the past two weeks, including anhedonia, depressed mood, sleep problems, fatigue, appetite changes, feelings of worthlessness, concentration difficulties, psychomotor changes, and suicidal thoughts. Each item is scored on a scale from 0 (“not at all”) to 3 (“nearly every day”), with higher total scores indicating more severe depressive symptoms;
- Seven item Generalized Anxiety Disorder (GAD-7) to detect generalized anxiety disorder (Spitzer et al., 2006). It consists of questions that evaluate core anxiety symptoms over the past two weeks, such as excessive worry, restlessness, irritability, and fear of impending doom. Each item is scored on a scale from 0 (“not at all”) to 3 (“nearly every day”), with higher total scores indicating more severe anxiety symptoms;
- Abbreviated (6 item) Post-Traumatic Checklist - Civilian Version (PCL-C) (Lang & Stein, 2005) is a shortened version of the original 17-item PCL-C, designed to assess symptoms of post-traumatic stress disorder (PTSD) in civilian populations. Each item is scored on a scale ranging from 1 (“not at all”) to 5 (“extremely”), indicating how much they have been bothered by each problem in the past month, with a score of 14 or higher suggestive of difficulties with post-traumatic stress.
- 10 item Connor–Davidson Resilience Scale (CD-RISC 10) (Campbell-Sills & Stein, 2007) is a questionnaire designed to assess an individual's resilience. Each item on a Likert scale from 0 (“not true at all”) to 4 (“true nearly all the time”), resulting in a total score ranging from 0 to 40, where higher scores indicate greater resilience.
- World Health Organization Disability Assessment Schedule (WHODAS) 12-item version (WHO, 2000) is a brief, standardized tool used to assess an individual’s disability and functioning across six domains. It measures difficulties experienced in the past 30 days, rated on a 5-point Likert scale (1 = no difficulty, 5 = extreme difficulty/cannot do) with higher scores indicating greater functional impairment; and
- Nine bespoke items designed to capture whether skills included in the intervention were being used by the participant (Appendix A).

All these tools, apart from the bespoke questionnaire, have been validated for use in India (De Man et al., 2021; Patel et al., 2022; Singh & Yu, 2010; WHO, 2000). Process data were collected about counselor training (e.g., pre–post knowledge test), supervision, and treatment (e.g., number of sessions, duration of sessions).

IDIs were conducted with treatment completers. Examples of domains covered in the IDIs include perceived changes in health status and social relationships since starting the intervention, and attribution of the change to the intervention. Additionally, these interviews explored the overall experience of the counseling process, challenges experienced during counseling, and barriers and facilitators to completing treatment.

Focus Group Discussions (FGDs) were conducted with counselors in Goa and IDIs were conducted with counselors in Mehsana at three different timepoints After their training, counselors participated in FGDs to determine satisfaction with the training, new skills learned, and confidence to respond to DV survivors. After delivering at least three sessions with one client, counselors took part in FGDs to ascertain satisfaction with training, needs from supervision, and the overall experience of delivering the intervention. Lastly, at the end of the treatment, FGDs were carried out with counselors to determine their satisfaction with intervention delivery including using the intervention manual and receiving supervision, challenges faced in counseling sessions and perceived changes in ability to respond to DV survivors. The qualitative interviews were audio-taped, transcribed verbatim, and translated into English.

The interviews were conducted by a team of trained researchers, with at least a graduate degree and/or field experience of the study context.

Serious adverse events were defined as physical/sexual violence causing injuries, requiring medical attention, or causing threat of harm to life; emotional violence causing threat of harm to life; the participant being forced out of her home and facing homelessness; suicide attempt; unplanned hospitalization; or death.

### Analysis

Process indicators of the treatment process and socio-demographic characteristics of the sample are presented as proportions and means as appropriate. The mean pre and post scores on the outcome scales (PHQ-9, GAD-7, CD-RISC, WHODAS, and PCL-C) were compared using the paired t-test. The paired t-test is used to compare two means in samples that are correlated and hence is suitable for studies with a “before-after” design.

Thematic analysis was used to analyze the qualitative data using NVivo 12. The transcripts for survivors and service providers were coded separately. All the coders were trained early career researchers with at least a graduate degree and/or field experience of the study context. Thematic coding was guided by the study objectives, forming an initial set of expected themes (e.g., “facilitators”). After deductively coding initial interview transcripts, we identified new codes inductively, refining the initial codebook before coding the complete dataset. Raw codes were generated from a section of the data by a pair of independent coders for each data set (DG & KM, MGP & SB, MGP & SH, AJ & SS), who then discussed and developed a common codebook. Similar codes were collapsed into inclusive categories, and clusters of related codes were organized under each category to generate themes. The themes were supported by linking excerpts from transcripts to demonstrate that themes were as close to the data as possible and reflected the words used by the participants themselves. These codebooks were then systematically applied to all interviews in the full dataset to generate a holistic picture of the acceptability, feasibility, and perceived effectiveness of this approach. Finally, data from IDIs and FGDs were compared to examine areas of convergence and divergence.

For the purpose of interpreting the findings, in the context of this study feasibility referred to any indicators of acceptability (How well the counselors and participants perceived and engaged with the intervention), implementation (The ease or difficulty of delivering the intervention), practicality (Whether the intervention can be delivered within the constraints of the setting), and preliminary impact (Early indications of whether the intervention can produce the intended outcomes).

### Ethics

Counseling sessions were conducted only at a mutually convenient safe place. Information leaflets were offered to the participants only if they believed that it was safe to take them home. We recruited only female counselors and case managers to deliver the intervention. Participants who were experiencing active IPV were referred to local services that could provide violence-specific support. All counselors were trained to identify medical/legal/social needs, provide preliminary support and information, and make supported referrals to reliable service providers through the case manager. All counselors were trained in risk management for suicide. Key staff from referral agencies were sensitized to the intervention to facilitate mutual trust and improve service responsiveness. The case manager was available to the client to accompany them to different service providers if needed. Potential participants who did not consent were given information about mental well-being and available services related to mental health and DV.

## Results

Table 1 summarizes the sociodemographic characteristics of the women. Compared to Mehsana, the participants in Goa were relatively older; and a greater proportion had completed formal education and were employed.

**Table 1.** Sociodemographic Profile of Participants.

	Goa	Mehsana
	*N* = 26	*N* = 12
	*N* (%)	*N* (%)
Mean age (SD) in years, range	46.9 (9.5), 32–65	37.8 (11.4), 25–60
Education status		
No formal education	2 (7.7)	4 (33.3)
Primary	6 (23.1)	4 (33.3)
Secondary	13 (50.5)	3 (25.0)
Higher secondary and above	5 (19.2)	1 (8.3)
Employment status		
Employed	13 (50.0)	5 (41.7)
Unemployed	3 (11.5)	1 (8.3)
Homemaker	10 (38.5)	6 (50.0)

Table 2 summarizes the treatment journey of all the participants in the study. In Goa, half of the participants were recruited through self-referral, referral by Anganwadi workers, and screening in a health facility. In Mehsana, all participants were recruited through referral by the *Atmiyata* Champions. While a greater proportion of participants in Goa dropped out before starting treatment, a greater proportion of participants in Mehsana completed treatment. While the greatest proportion of participants in Goa chose to receive the intervention in their homes, the greatest proportion of participants in Mehsana preferred to receive the intervention in the *Atmiyata* Champions’ homes. On an average, participants received five sessions at both sites and some participants at both sites continued to experience violence (20% in Goa and 25% in Mehsana) while they were receiving the intervention.

**Table 2.** Treatment Journey of Study Participants.

	Goa *N* = 26 *N* (%)	Mehsana *N* = 12 *N* (%)
Referral pathway		
Self	5 (19.2)	
Screened at health facility	4 (15.4)	
Anganwadi	4 (15.4)	
Workplace	3 (11.5)	
Other participant	3 (11.5)	
Village organization	2 (7.7)	
Self-help group	3 (11.5)	
Host institution staff	2 (7.7)	
Atmiyata Champion/community gatekeeper		12 (100)
Treatment status		
Did not start treatment	6 (23.1)	2 (16.7)
Dropped out after starting treatment	4 (15.4)	1 (8.3)
Planned discharge before completing six sessions	3 (11.5)	0 (0)
Planned discharge after completing six sessions	13 (50.0)	9 (75.0)
Referral status		
Did not require supportive referral	24 (92.3)	12 (100)
Local organization for shelter	1 (3.8)	0 (0)
District hospital for treating injuries	1 (3.8)	0 (0)
Location of intervention delivery		
Participant's home	10 (45.5)	3 (25.0)
Participant's friend's or other family member's home	4 (18.2)	2 (16.7)
Public space	4 (18.2)	1 (8.3)
Participant's home and public space	4 (18.2)	0 (0)
Atmiyata Champion's home	0 (0)	6 (50.0)
Mean number of sessions attended (SD)	5.3 (2.2)	5.7 (0.9)
Experienced violence during the treatment period	6 (20.0)	3 (25.0)

In all we had 209 ratings of sessions reviewed during supervision—54 (25.8%) by supervisor, 85 (40.7%) by the counselor whose session was being reviewed, and 70 (33.5%) by peers. Appendix B compares the mean scores on the various items of the scale between the three phases of the study. There was a substantial variation in competency acquisition based on the type of skills measured. Overall, the counselors did not achieve an average of 4 on any of the competencies measured. The following trends were observed in competency acquisition: (a) counselors plateaued (no substantial change over the three phases) at a reasonable level of competency from the outset (e.g., nonverbal communication, exploration and normalization, reflection of thoughts and feelings), (b) counselors plateaued (no substantial change over the three phases) at a low level of competency from the outset (e.g., connection to social functioning, involving family), (c) substantial initial improvement followed by plateauing (e.g., rapport building, warmth and empathy, exploration of social support), (d) substantial improvement only in the later stages (e.g., suicide risk assessment and safety planning), (e) gradual improvement throughout the study (e.g., verbal communication), (f) deterioration of competencies (e.g., confidentiality).

For the GAD-7, PCL-C, and WHODAS, there was a statistically significant improvement in scores from baseline to midline, midline to endline, and baseline to endline. For PHQ-9 and CD-RISC there was a statistically significant improvement in scores from baseline to midline and baseline to endline, and not midline to endline. Table 3 summarizes the comparison of outcomes at the various assessment timepoints.

**Table 3.** Outcome Evaluation at the Baseline, Midline, and Endline.

		Goa	*p* ^a^	Mehsana	*p* ^a^
PHQ-9	Baseline	9.4 (5.2)	B vs. M; *p = *.0001	11.8 (3.4)	B vs. M; *p = *.06
	Midline	3.2 (3.5)	M vs. E; *p = *.59	8.1 (4.8)	M vs. E; *p = *.17
	Endline	3.3 (3.1)	B vs. E; *p < *.001	3.4 (7.4)	B vs. E; *p = *.01
GAD-7	Baseline	6.8 (4.5)	B vs. M; *p < *.001	11.1 (4.0)	B vs. M; *p = *.008
	Midline	2.6 (2.8)	M vs. E; *p = *.30	5.9 (4.5)	M vs. E; *p = *.005
	Endline	2.3 (3.0)	B vs. E; *p = *.0001	0 (0)	B vs. E; *p < *.001
PCL-C	Baseline	17.4 (4.6)	B vs. M; *p = *.001	21.6 (4.3)	B vs. M; *p = *.02
	Midline	12.9 (5.7)	M vs. E; *p = *.032	16.6 (3.6)	M vs. E; *p = *.02
	Endline	10.2 (4.1)	B vs. E; *p < *.001	11.8 (5.1)	B vs. E; *p = *.002
CD-RISC 10	Baseline	17.0 (7.9)	B vs. M; *p = *.05	19.7 (4.8)	B vs. M; *p = *.002
	Midline	22.8 (7.9)	M vs. E; *p = *.0007	27.6 (3.4)	M vs. E; *p = *.17
	Endline	30.2 (6.0)	B vs. E; *p = *.0001	30.3 (4.2)	B vs. E; *p < *.001
WHODAS	Baseline	7.8 (6.7)	B vs. M; *p = *.0005	16.7 (6.1)	B vs. M; *p = *.001
	Midline	3.4 (3.7)	M vs. E; *p = *.02	6.1 (4.5)	M vs. E; *p = *.02
	Endline	2.5 (3.3)	B vs. E; *p = *.0006	2.2 (2.6)	B vs. E; *p < *.001

a Paired *t*-test.

**Figure 2.**
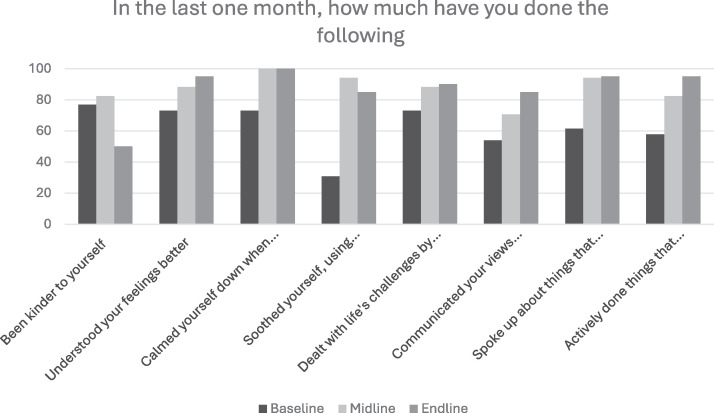
Comparison of Strategies Used by Participants at Three Assessment Timepoints (Goa).

**Figure 3.**
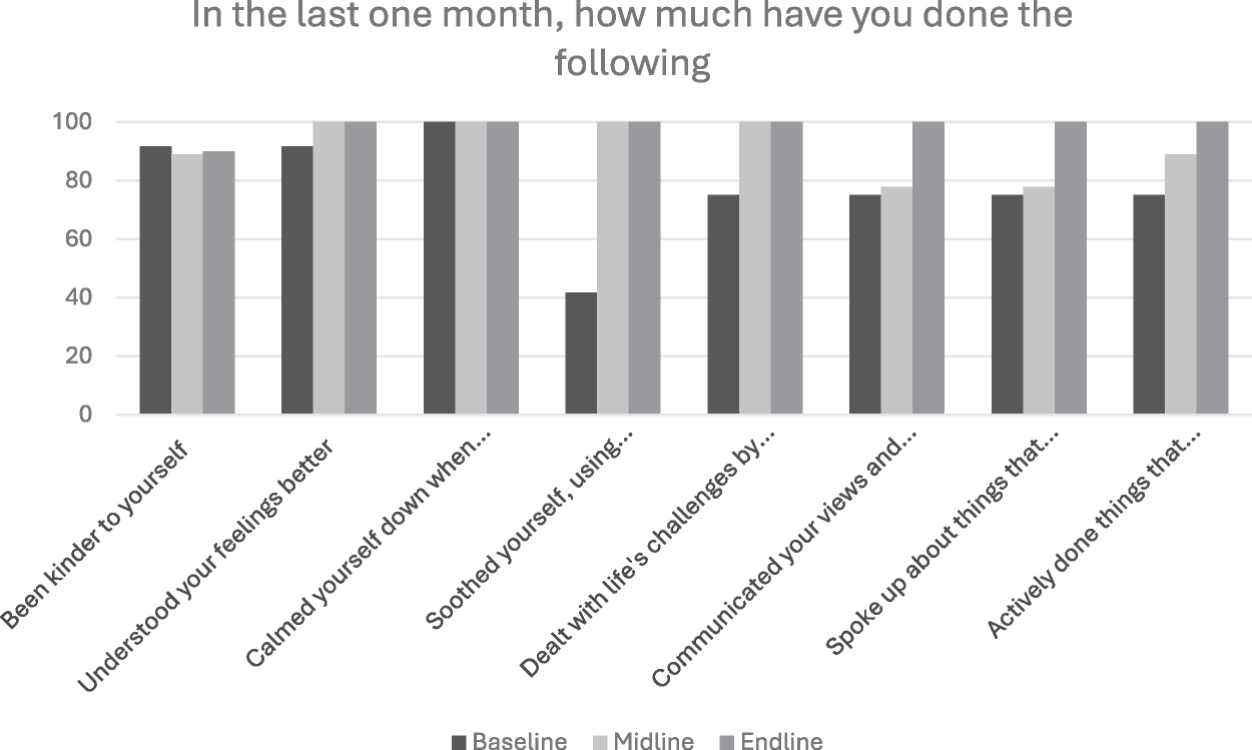
Comparison of Strategies Used by Participants at Three Assessment Timepoints (Mehsana).

We compared the utilization of various intervention strategies between baseline, midline and endline (Appendix C; Figures 2 and 3). In Goa, the following key trends were seen in the proportion of participants who were using the various strategies: (a) increase over the three time points (e.g., understood your feelings better, dealt with life’s challenges by breaking them down and tackling them one step at a time, communicated your views and feelings to your family members or resolved conflict with family members, actively done things that improve your mood like doing joyful activities or activities that give you a sense of accomplishment/success), (b) steep increase followed by a plateau (e.g., Calmed yourself down when angry, spoke up about things that bother you, expressed feelings, sought help), and (c) initial increase followed by a drop (e.g., Been kinder to yourself, soothed yourself, used breathing techniques, or progressive muscle relaxation).

In Mehsana, the following key trends were seen in the proportion of participants who were using the various strategies: (a) increase over the three time points (e.g., communicated your views and feelings to your family members or resolved conflict with family members, spoke up about things that bother you, expressed feelings, sought help, actively done things that improve your mood like doing joyful activities or activities that give you a sense of accomplishment/success), and (b) steep increase followed by a plateau (e.g., soothed yourself, using breathing techniques or progressive muscle relaxation, dealt with life’s challenges by breaking them down and tackling them one step at a time, understood your feelings better).

**Figure 4.**
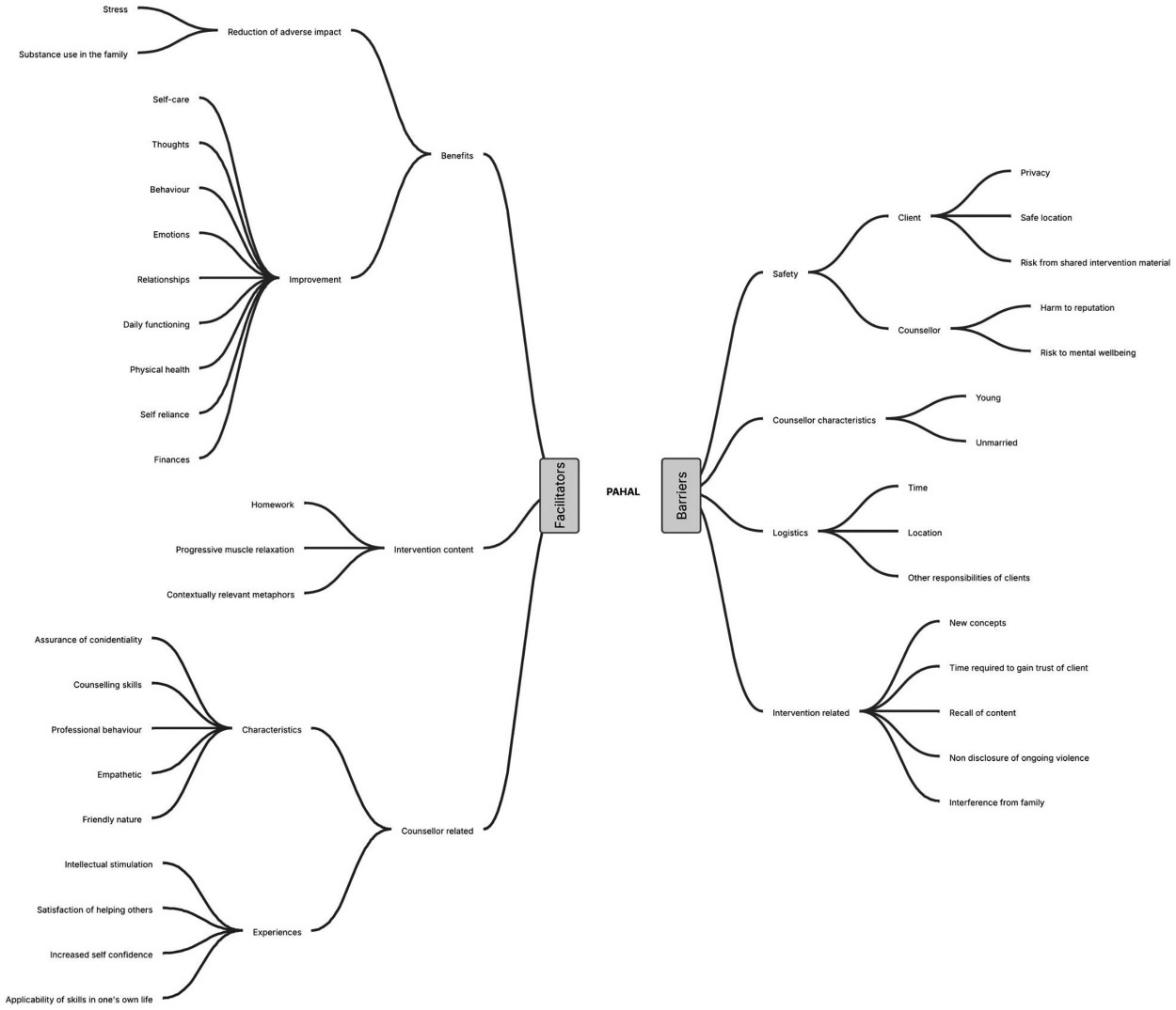
Barriers and Facilitators to Delivering PAHAL.

We conducted IDIs with 28 participants and 10 counselors; and two FGDs with counselors. The following section summarizes the triangulated findings from the nested qualitative study organized as barriers and facilitators to intervention (Figure 4) for IPV from the perspectives of the counselors and participants.

### Facilitators

The facilitators of the intervention included the benefits accrued by the clients, the content of the intervention, characteristics of the counselors and the positive experiences of the counselors themselves.

#### Benefits to the Clients

Both counselors and participants reported benefits of the intervention to the participants. These included improvement in various aspects of their lives and reduction in the adverse impacts of the IPV. Broadly, the changes they experienced after counseling included the following:

- Improvement in thoughts and behavior. This included ceasing to think negatively, becoming active and independent, increased interest in work and home life, enhanced self-confidence, “mind becoming lighter,” generally feeling better, and becoming firm in decision-making;
  And now I feel like going somewhere. I do not take help from others, on my own I do it and I go. I do not even take help from my husband. (Participant, Goa)
- Improvement in managing difficult emotions, primarily in the form of controlling anger and overcoming fear;
  The fear that was there, it reduced. If we think of what will happen in the future, then we tend to feel more troubled. So that reduced a little. (Participant, Goa)
  When [counselor] told me to sit and listen to what she says, she told me to keep things calm, keep mind calm and handle everything peacefully. (Participant, Mehsana)
- Improvement in relationships through improved communication and enhanced ability to share experiences and feelings with husband, relatives, children, and others;
  I did not want those people only [did not want to interact], but now it is not like that, now I get involved, I laugh, I talk with everyone, I call them. Earlier I used to not call them much, what to keep telling them about me, like this I used to feel, that is why I used to not call anyone much, now I do, I talk to them. (Participant, Goa)
  I hated everyone, even my children, but after meeting [counselor] I can talk with them. very politely and sit with them and talk peacefully (Participant, Mehsana)
- Better self-care, such as developing new hobbies, introduction of physical activity into their daily routines, improved diet, and paying better attention to themselves;
- Other positive changes, such as improved daily functioning, better physical health, greater self-reliance, and improved financial status; and
- Reduction in the adverse associations such as stress and substance use in the family.
  Yes, I would not work or I will sleep or I would go somewhere or I feel suicidal or I used to have bad thoughts, now I have all good thoughts. I want to live peacefully now. Let God take care of everything. Now everything is good now I am able to work. (Participant, Mehsana)

#### Intervention Content

The counselors reported that some participants did do their homework and found strategies such as progressive muscle relaxation particularly useful to practice between sessions.

PMR– my yesterday’s client, her six sessions got over, in first five she did not do anything but for six days she did PMR continuously and with that there was great difference. If she sat down then she was not able to get up without anyone’s help, and yesterday she was showing me that, “see I am able to get up without any help”, because PMR helped her … (Counselor, Goa)

Additionally, during sessions, using simple and relatable metaphors were found to be especially useful.

And I observed that when we included role plays and examples like the example of nature—then daily functioning, what they do daily? How they do it? When that was given to them that time they started understanding it better “Yes when I—suppose if we gave example of tree, if the roots of the trees are strong then all flowers, fruits will be there, even if rain comes, wind blows, it will remain strong similarly after giving one example “Yes, even I should remain confident only then I can deal with the active violence” like this they were getting the confidence. (Counselor, Goa)

All those 16 skills were so useful. One of them is named boundary setting - that I will not tolerate [the violence]. I liked it so much because at some places when we are supposed to say “no” we have to say no and that’s necessary. (Counselor, Mehsana)

#### Counselor-Related Factors

These included the characteristics of the counselors and counseling relationship, as well as the positive experiences of the counselors.

**Counselor Characteristics.** Other facilitators related to the program included the fact that the counselor was not someone known to the participant and a resulting perception of enhanced confidentiality; and characteristics of the counselor, such as good counseling skills, professional behavior, empathy (understood and supported me), and friendly attitude.

She [counselor] is friendly, never gets fed up, she always used to come smiling, she was not feeling bored, she has a good nature, she tried to understand me a lot, earlier also—she used ask me about my well-being, like “how are you?” We do not have any relation, still she used to, that was a big thing. (Participant, Goa)

It was a lot supportive for me. Because now I told isn’t it, I cannot tell neighbors, cannot tell the family, cannot even tell the friends circle. Because then it spreads everywhere. But there is such a person who only listens to me, understands me and what decisions I take in my life, what will happen because of it, when we talk about it and share it. (Participant, Goa)

**Counselor Experiences.** The counselors largely reported positive experiences during intervention delivery, such as intellectual stimulation at learning something new and satisfaction in helping those in need. Some strategies that they found to be useful included refreshing their memory by referring to the manual before the session, helping the participants to practice self-kindness, and using the Feelings Wheel (a delivery aid to help the participant reflect on their emotions). Finally, counselors reported feeling that the new competencies they had acquired helped them, as their self-confidence increased and they could use some of the skills in their personal lives.

Yes, confidence increases. And what happens now is if we go to talk normally with someone we automatically use these skills without realizing it. (Counselor, Mehsana)

### Barriers

These included the concerns related to the safety of the client as well as the counselor, counselor characteristics, logistics, and intervention related factors.

#### Safety

**Safety of the Client.** One key concern was related to the safety of the participant and the counselor. This included practical challenges, such as finding a safe and private location for sessions, maintaining participant's privacy, and risks (e.g., of further violence) due to material shared with participants.

Yes, violence can be caused by the husband, if we go to their house. Why does this sister [counselor] come often? She will often go to teach my wife something, there will be more fights between us. If that comes to their [husband's] mind then the violence will increase. (Counselor, Mehsana)

It is also difficult because when we are talking with the beneficiary if some other person came and asks us about what we are filling [intervention materials] then it becomes difficult to handle the situation. What if the third person came to know about it, then? (Counselor, Mehsana)

Sometimes while delivering the session in the temple, people used to disturb uh… people were questioning, why have you come? There we had to face the challenges because you know how people from village are. They do not have that knowledge when you say counseling, what are you doing? When you say counseling that time they say there is some problem, like this they say but we had to think from client’s perspective that where it is safe for her, where session can be delivered? (Counselor, Goa)

**Safety of the Counselor.** While there were concerns about increased risk of harm to the participants, the counselors were also worried about their own safety, the occupational cost of continuing such work, harm to their reputation, and risk to their well-being because of the work with IPV and trauma.

When we do these activities [counseling], we also have to face challenges from home. Mother-in-law and others would say where are you loitering and avoiding household chores. These we also have to face. (Counselor, Mehsana)

If we [counselor] call her [participant] to our home then her husband does not trust what she is going to learn. What is that sister [counselor] teaching her? So, living in a rural area we have to make sure that our image doesn’t get spoiled. (Counselor, Mehsana)

When she [participant] was telling me her story that time, they were expressing their emotions and as a new counselor, at the beginning I faced many challenges to control my emotion, I found it difficult, when they were crying that time somewhere I too used to shed some tears but after a few cases I was fine. (Counselor, Goa)

#### Counselor Characteristics

From the participants’ perspective, some of the challenges were the counselors’ young age, and unmarried status.

But that girl [counselor] was unmarried, right? I have two children. So if she was somebody of my age, if she was married, then I could have spoken to her openly. Right? She was very young compared to me. And I am a mother of two children. So that is why I felt a little awkward. [Participant, Goa]

#### Logistical Challenges

Common barriers included finding an appropriate time and safe location for the counseling, and being preoccupied with other tasks and responsibilities.

Difficulties as in, at times, my husband would be at home, children’s exams, I wouldn't get time, my younger child fell sick, he got fever. One by one, there came difficulties. And then I did not get to take it [attend counseling]. [Participant, Goa]

#### Intervention-Related Challenges

From the counselors’ perspective, they experienced challenges related to the intervention manual and with certain aspects of working with participants. The intervention-related challenges included requiring time to understand new concepts introduced in the training and hence finding the intervention hard to use in early stages. For example, they reported some difficulty in setting goals in session 1 with clients, finding it difficult to maintain the flow of sessions, and disruption to the flow of the session resulting from the use of information leaflets within the session. The participant-related challenges included the time taken to gain the participants’ trust and for them to open-up, participants not remembering the content covered, participants hiding active violence from counselors, questions from and involvement of family members, and scheduling challenges.

## Discussion

This paper presents the acceptability, feasibility, and preliminary impact testing of an evidence-informed and contextually appropriate psychosocial intervention to be delivered by nonspecialist workers to address CMDs among survivors of IPV in India. The process data indicated that it is reasonably feasible to identify, recruit, and retain the target population in the program. There is good acquisition of skills and utilization of psychosocial strategies by the recipients through the course of the counseling. Finally, the trends of change for all outcomes were in the expected direction, indicating early evidence of potential effectiveness. Overall, the intervention was feasible to be delivered by nonspecialist workers and perceived to be useful by both the counselors and participants. With the work in Mehsana, we have been able to demonstrate the feasibility and acceptability of training existing community mental health workers to deliver another psychosocial intervention in addition to the one they were originally trained to deliver. Along with other evidence that demonstrates that nonspecialist workers can effectively deliver two different psychosocial interventions (Jordans et al., 2019; Nadkarni et al., 2017; Weobong et al., 2017), this has important implications for low resource settings that cannot afford condition-specific counselors.

There is growing evidence that psychosocial interventions may reduce the impact of IPV in low- and middle-income settings. For example, in such settings, psychosocial interventions reduced any form of IPV by 25% at the longest follow up; more specifically reducing physical IPV by 27% and sexual IPV by 23% (Turner et al., 2020). Another example of task-sharing with nonspecialists is research conceptualizing peer support work in relation to IPV, which suggests that peer support can offer holistic and comprehensive emotional and practical assistance while emphasizing survivor agency and autonomy, which traditional IPV response approaches might be unable to do (Osborn et al., 2024).

Our study adds to the limited evidence base for potentially scalable task shared psychosocial interventions for mental health problems for historically neglected population groups due to their high-risk status as IPV survivors (Keynejad et al., 2020). There is substantial evidence that the mental health of survivors of IPV is most effectively supported through personalized interventions grounded in their culture and context, which also honour the complexity of each woman's situation (Paphitis et al., 2022). Despite this, evidence of effectiveness of such interventions in LMICs is limited, with only four studies from India (Paphitis et al., 2022). However, unlike our intervention, none of the interventions tested in the trials from India provided individually focused psychosocial treatment for CMDs among IPV survivors. Hence, our study and the findings reported in this paper are an important step toward the development of a contextually relevant intervention that is suited for LMICs and is responsive to the mental health needs of survivors of IPV due to its modular design and inclusion of options for using varied evidence-based skills.

We would like to acknowledge some limitations of our study which would influence the interpretation of the findings. Since we used an uncontrolled treatment cohort with pre-post evaluation, it is difficult to determine whether the observed changes are due to the Pahal intervention itself or to other factors such as natural recovery, other external events occurring during the study period that could have influenced participants’ mental health, and regression to the mean for participants with extreme scores on the various measures. We used convenience sampling to identify participants, and this can introduce selection bias which limits the generalizability of the findings. The exclusion of women experiencing severe and high frequency violence in Goa limits the scope of our findings. Finally, the study relies on self-reported data, which can be subject to recall bias and social desirability bias.

One of the key strengths of our study lies in the triangulation of quantitative data (outcome and process data) and qualitative findings (from two different sources) across two distinctive implementation sites in rural and semiurban India. This approach allows for a more holistic understanding of the feasibility and acceptability of the *Pahal* intervention's implementation potential. Although the quantitative mental health and related outcomes changed in the desired direction, the absence of a control arm precludes any conclusion about the role of the intervention in creating these changes. However, the positive impact of the intervention on several outcomes was corroborated by participants, who reported perceived benefits in qualitative interviews. The strong endorsement by intervention participants of the purported mechanisms that we incorporated into the treatment design constitutes a robust rationale for further study of *Pahal*'s effectiveness in a randomized controlled trial.

Some key implications of our findings relate to future definitive testing of *Pahal. L*essons learned from this project have informed the following considerations and potential changes: revisions to the eligibility criteria (e.g., inclusion of those experiencing ongoing violence); making the treatment trauma-focused; and enhancing protocols to ensure privacy, confidentiality and safety, as these were crucial concerns for both the counselors and the participants. Finally, there were variations in how we implemented the program at the two sites, highlighting the importance of being responsive to contextual conditions.

Some potential policy and practice implications that arise from our study are as follows. First, healthcare systems could expand access to IPV support by training nonspecialist workers to deliver interventions such as Pahal. Implementing interventions within existing community programs, as done in Mehsana with Atmiyata, can increase reach and acceptability, especially for women who may not seek help from formal institutions due to stigma, patriarchal norms, or lack of access to services. Our study emphasizes the importance of tailoring interventions to the specific cultural context and needs of the target population. Given the high rates of co-occurring mental health problems and IPV, interventions should specifically address mental health needs through components such as problem-solving, cognitive restructuring, and intensive advocacy. Finally, there are various reasons why women experiencing IPV may not seek help, including patriarchal social norms, stigma, and lack of trust in formal institutions. Interventions should address these barriers by promoting awareness, challenging harmful norms, and building trust in community-based support systems.

## Conclusion

The key outcome of our study is a better understanding of the acceptability and feasibility of *Pahal* and its potential to effect change. If found to be effective in a definitive RCT, *Pahal* has the potential to address the specialist human resource-related barriers to scaling effective interventions to meet the needs of IPV survivors in low resource settings.

## Appendices

**Appendix A.** Mechanisms of Change.

In the last one month…How much have you done the following…				
No.	Construct (not to be mentioned in the survey)	Definition	Rate 0–4 (0 = not at all; 2 = somewhat; 4 = all the time)
1	Exercised self-compassion	Being kinder to yourself (e.g., speaking to yourself nicely when you make a mistake)	
2	Developed knowledge of your emotions	Understanding your feelings better (e.g., with more detail)	
3	Controlled your anger	Being able to calm yourself down when angry	
4	Practiced relaxation techniques	Soothing yourself, using breathing techniques, or progressive muscle relaxation	
5	Set goals and solved problems	Dealing with life’s challenges by breaking them down and tackling them one step at a time	
6	Used negotiation, boundary setting, assertive communication, and conflict resolution	Communicated your views and feelings to your family members or resolved conflict with family members	
7	Disclosed/ sought help	Speaking up about things that bother you and expressing feelings, seeking help	
8	Activated your behavior	Actively doing things that improve your mood like doing joyful activities or activities that give you a sense of accomplishment/success	
9	Used a safety plan (to be added ONLY in the midline and endline surveys)	Looked at your safety plan and practiced coping strategies from it to practice when you had thoughts of hurting yourself

**Appendix B.** Competency Evaluation During Peer Supervision.

Skill	Months 1–2 *M* (SD)	Months 3–4 *M* (SD)	Months 5–6 *M* (SD)
*How skills*			
Nonverbal communication	2.7 (0.5)	2.9 (0.6)	3.0 (0.5)
Verbal communication	2.4 (0.8)	2.7 (0.7)	2.9 (0.7)
Confidentiality	2.8 (0.5)	2.1 (1.3)	1.7 (1.6)
Rapport building	2.3 (0.8)	2.9 (0.6)	3.1 (0.4)
Exploration and normalization	2.1 (0.9)	2.3 (0.9)	2.2 (1.1)
Warmth and empathy	1.9 (0.8)	2.7 (0.7)	2.8 (0.6)
Suicide risk assessment and safety planning	1 (0)	1 (1.3)	2.3 (1.2)
Reflection of thoughts and feelings	2 (0)	1.9 (0.9)	1.9 (1.2)
Connection to social functioning	1.8 (1.1)	1.7 (1.0)	2.1 (1.1)
Exploration of social support	1 (0)	1.8 (1.0)	2.2 (1.2)
Involving family	1 (0)	1.5 (0.9)	1.4 (0.5)
Collaborative goal setting	1.7 (0.9)	2.1 (1.0)	2.2 (1.1)
Promoting realistic hope	1.9 (0.8)	2.2 (0.9)	2.4 (0.9)
Incorporating coping skills	1.5 (1.0)	2.0 (1.0)	2.4 (1.1)
Psychoeducation	1.8 (1.4)	1.6 (1.2)	1.9 (1.3)
Elicitation of feedback	1.4 (1.5)	2 (0.9)	1.9 (1.3)
*What skills*			
Managing difficult emotions	2 (0.8)	2 (1.0)	2.1 (1.1)
Anger management	2.3 (0.5)	2.1 (1.0)	2.1 (1.0)
Self-soothing and body awareness	2.4 (0.7)	2.1 (1.0)	2.4 (1.0)
Self-kindness	1.6 (0.9)	2 (1.1)	2.2 (1.2)
Problem solving	2 (0.8)	2 (1.0)	2.4 (1.0)
Goal setting	1.6 (0.9)	1.9 (0.9)	2.1 (1.2)
Assertive communication	2 (1.4)	1.3 (1.1)	2.6 (0.9)
Negotiation	0 (0)	1.3 (1.0)	2.7 (0.7)
Boundary setting	0 (0)	1.8 (2.1)	2.4 (1.8)
Conflict resolution	0 (0)	0.5 (0.7)	2.3 (1.9)

**Appendix C.** Comparison of the Strategies Used by the Participants.

	Goa			Mehsana		
In the last one month, how much have you done the following:	Baseline (*n* = 23)	Midline (*n =* 17)	Endline (*n =* 20)	Baseline (*n =* 12)	Midline (*n =* 9)	Endline (*n =* 10)
Been kinder to yourself	20 (76.9)	14 (82.4)	10 (50.0)	11 (91.7)	8 (88.9)	9 (90.0)
Understood your feelings better	19 (73.1)	15 (88.2)	19 (95.0)	11 (91.7)	9 (100.0)	10 (100.0)
Calmed yourself down when angry	19 (73.1)	17 (100)	20 (100)	12 (100.0)	9 (100.0)	10 (100.0)
Soothed yourself, using breathing techniques, or progressive muscle relaxation	8 (30.8)	16 (94.1)	17 (85.0)	5 (41.7)	9 (100.0)	10 (100.0)
Dealt with life’s challenges by breaking them down and tackling them one step at a time	19 (73.1)	15 (88.2)	18 (90.0)	9 (75.0)	9 (100.0)	10 (100.0)
Communicated your views and feelings to your family members or resolved conflict with family members	14 (53.9)	12 (70.6)	17 (85.0)	9 (75.0)	7 (77.8)	10 (100.0)
Spoke up about things that bother you, expressed feelings, sought help	16 (61.5)	16 (94.1)	19 (95.0)	9 (75.0)	7 (77.8)	10 (100.0)
Actively done things that improve your mood like doing joyful activities or activities that give you a sense of accomplishment/success	15 (57.7)	14 (82.4)	19 (95.0)	9 (75.0)	8 (88.9)	10 (100.0)
